# Correlation between Group B Streptococcal Genotypes, Their Antimicrobial Resistance Profiles, and Virulence Genes among Pregnant Women in Lebanon

**DOI:** 10.1155/2009/796512

**Published:** 2010-02-02

**Authors:** Antoine Hannoun, Marwa Shehab, Marie-Therese Khairallah, Ahmad Sabra, Roland Abi-Rached, Tony Bazi, Khalid A. Yunis, George F. Araj, Ghassan M. Matar

**Affiliations:** ^1^Department of Obstetrics and Gynecology, Faculty of Medicine, American University of Beirut, 11072020 Beirut, Lebanon; ^2^Department of Microbiology and Immunology, Faculty of Medicine, American University of Beirut, 11072020 Beirut, Lebanon; ^3^Department of Pediatrics and Adolescent Medicine, Faculty of Medicine, American University of Beirut, 11072020 Beirut, Lebanon; ^4^Department of Pathology and Laboratory Medicine, Faculty of Medicine, American University of Beirut, 11072020 Beirut, Lebanon

## Abstract

The antimicrobial susceptibility profiles of 76 *Streptococcus agalactiae* (Group B Streptococci [GBS]) isolates from vaginal specimens of pregnant women near term were correlated to their genotypes generated by Random Amplified Polymorphic DNA analysis and their virulence factors encoding genes *cylE, lmb, scpB, rib*, and *bca* by PCR. Based on the distribution of the susceptibility patterns, six profiles were generated. RAPD analysis detected 7 clusters of genotypes. The *cylE* gene was present in 99% of the isolates, the *lmb* in 96%, *scpB* in 94.7%, *rib* in 33%, and *bca* in 56.5% of isolates. The isolates demonstrated a significant correlation between antimicrobial resistance and genotype clusters denoting the distribution of particular clones with different antimicrobial resistance profiles, entailing the practice of caution in therapeutic options. All virulence factors encoding genes were detected in all seven genotypic clusters with *rib* and *bca* not coexisting in the same genome.

## 1. Introduction

Neonatal group B streptococcus (GBS) infection ranging from 0.5 to 2 per 1000 live birth is the result of vertical transmission to the infant during delivery in colonized mothers [1]. Attempts at identifying GBS colonized pregnant women near term by assessing the risk factors for GBS, failed to diagnose all cases [2]. Universal screening of all pregnant women for GBS at 35–37 weeks of gestation is currently recommended by the Centers for Disease Control and Prevention (CDC) in the USA [2]. 

 Despite the variation in the adherence to CDC recommendations among different centers around the world, antimicrobial prophylaxis, offered to women colonized with GBS intrapartum (after onset of labor or after rupture of membranes), lead to more than 70% reduction in early-onset GBS infection in neonates [3]. However, due to the variation in the rate of GBS colonization during pregnancy [4], the emergence of resistant colonizing strains [5] and the virulence potential of these strains, intrapartum screening of GBS genotypes [6] along with their antimicrobial resistance profiles, and virulence encoding genes become desirable. 

 Fortunately, GBS resistance to penicillin has not been reported so far [7]; however, rare GBS clinical strains with reduced sensitivity for penicillin have been recorded [8]. On the other hand, early studies on GBS isolates from pregnant women, showed antimicrobial resistance as high as 18%, 8%, and >80% for erythromycin, clindamycin, and tetracycline respectively [9]. 

 The severity of neonatal disease in GBS infections could be determined mostly by a number of virulence factors encoded among others by the *cps *gene cluster coding for the capsule [10], the *scpB* gene coding for surface enzyme ScpB (a C5a peptidase) which causes impairing of neutrophil recruitment and binds fibronectin to promote bacterial invasion of epithelial cells [11], the *bca* gene coding for alpha-C protein, a surface protein that helps the bacteria to enter the host cells [12], the *lmb* gene coding for lmb (laminine-binding protein), a surface protein that plays a role in invasion of damaged epithelium [13], the *cylE* gene coding for *β*-hemolysin, a toxin that plays a role in tissue injury and systemic spread of the bacteria and contributes to meningitis [14], and the *rib* gene encoding the surface Rib protein mostly present in invasive strains [15]. Other virulence factors involved in the process of GBS pathogenesis include beta-C protein which is encoded by *bac* gene. Beta-C protein function is comprised of interaction with IgA-Fc portion causing the inhibition of phagocytosis [16] and binding Factor-H to maintain its role in inhibiting the complement activation via the alternative pathway [17]. Other important virulence factors of GBS are the Fibrinogen-Binding proteins: FbsA and FbsB. FbsA is a surface protein encoded by *fbsA* gene. It protects the pathogen from opsonophagocytosis, and promotes its adherence to epithelial cells and to the human brain microvascular endothelial cells (HBMEC) thus helping it to cross the blood brain barrier and developing meningitis [18, 19]. FbsB protein, encoded by *fbsB* gene, is also a surface protein that helps in GBS invasion of the epithelial cells [20]. Finally, cell-surface-associated protein (CspA) is a surface protein encoded by *cspA* gene. It is involved in maintaining the pathogen survival in the host by escaping the immune system [21]. 

 In this study, genotypes were correlated to some of the virulence genes and the antimicrobial susceptibility profiles. This information is useful to identify particular GBS strains with high virulence potential, with resistance to routinely administered antimicrobial agents, and possibly linked to particular geographical areas.

## 2. Materials and Methods

### 2.1. Bacterial Isolates

Seventy six GBS isolates were cultured from specimen taken from the vaginas of pregnant women between 35 and 37 weeks of gestation, attending private clinics in two tertiary care centers in Beirut, Lebanon, between October 2007 and July 2008. The first center (A) is located in the western part of the urban city of Beirut, and the second one (B) in the eastern part of Beirut. These two hospitals serve two different populations, in the sense that patients attending the hospitals come from 2 different geographical areas, with very minimal intermingling between them. However, the local health care systems of the two areas are similar, and patients have similar antibiotics consumption habits. The 76 GBS isolates consisted of 47 (61.8%) isolates obtained from the first tertiary care center and 29 (38.2%) isolates obtained from the second tertiary care center.

### 2.2. Identification

Isolates were identified using conventional methods on the basis of colonial morphology, Gram staining, haemolysis, and latex agglutination test with specific antisera using the Slidex Strepto Plus (BioMerieux, Marcy L'Etoile; France).

### 2.3. Antimicrobial Susceptibility Testing

Antimicrobial susceptibility testing was performed by the disk-diffusion (Kirby-Bauer) method on Mueller-Hinton agar (Difco Laboratories, Detroit, Michigan) supplemented with 5% sheep blood, using suspensions of 0.5 MacFarland from fresh bacterial cultures. The test was done using the following antimicrobial agents: penicillin, cefepime, ceftriaxone, chloramphenicol, clindamycin, erythromycin, levofloxacin, and tetracycline. Results were interpreted according to the Clinical Laboratory Standard Institute (CLSI) [22].

### 2.4. Total DNA Extraction

DNA was extracted from all isolates using the illustra bacteria genomicPrep Mini Spin Kit (GE Healthcare UK Ltd, Buckinghamshire, England) according to the manufacturer's instructions.

### 2.5. Polymerase Chain Reaction (PCR)

Total DNA of the 76 GBS isolates was used to amplify five virulence factors encoding genes: *cylE* [23], *rib* [24], *lmb* [25], *bca* [25], and *scpB* [26] by PCR using specific primers. Standard PCR conditions were used to amplify *cylE*, *lmb*, and *scpB* [23], *rib* [24], and *bca* gene [25]. 

 PCR reactions were performed in the Sprint, Thermo Electric thermal cycler. Amplicons were subjected to electrophoresis on 2% agarose (Sigma) gels in 1 × Tris-Borate-EDTA buffer pH 8.3 (Tris base 0.089 M, Boric acid 0.089 M, and EDTA 0.002 M) at 120 V for 45 minutes. 

 Agarose gels were stained with ethidium bromide (Sigma) and photographed using a UV-transilluminator and an Olympus digital camera with Digi-Doc it Program.

### 2.6. Genotyping

Genotyping using Random Amplified Polymorphic DNA (RAPD) analysis was performed on all the isolates to determine strain diversity. RAPD was performed with the Ready-To-Go RAPD Analysis Beads kit (GE Healthcare UK Ltd, Buckinghamshire, England) and the GBS 2 primer [6], using Sprint, Thermo Electric thermal cycler. A dendrogram was generated for the RAPD patterns using the BIONUMERICS software. (Applied Maths, Saint-Martens-Latem, Belgium.)

## 3. Results

### 3.1. Bacterial Isolates Distribution and Antimicrobial Susceptibility

Antimicrobial susceptibility testing showed the following: all GBS isolates were susceptible to penicillin G, cefepime, ceftriaxone, and levofloxacin. The following percentages of isolates, 4%, 11.8%, 15.8%, and 86.8%, were resistant to chloramphenicol (CHL), clindamycin (CLI), erythromycin (ERY), and tetracycline (TET) respectively. Six antimicrobial resistance (AR) profiles of isolates were detected: A (resistant to CLI, ERY, CHL, and TET; 4.0%), B (resistant to CLI, ERY, and TET; 6.6%), C (resistant to CLI and TET; 1.3%), D (resistant to ERY and TET; 5.3%), E (resistant to only TET; 69.7%), and F (susceptible to all; 13.2%).

### 3.2. RAPD Analysis

**Figure 1:**
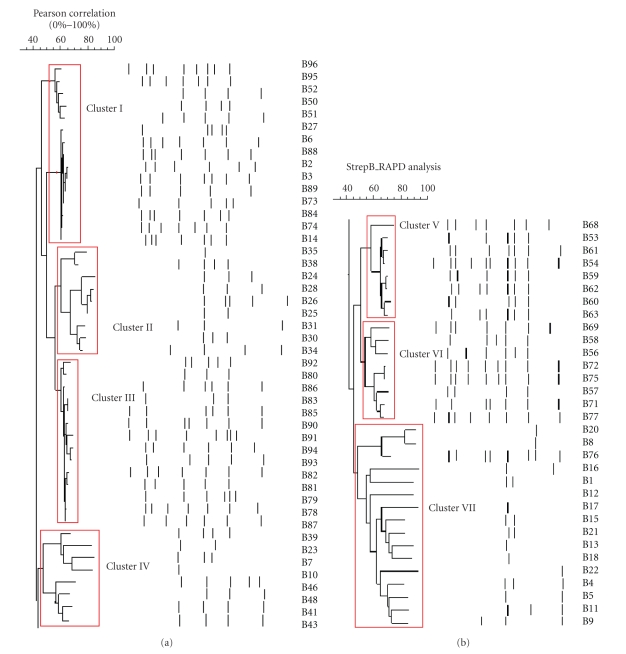
Dendrogram of the random amplified polymorphic DNA (RAPD) analysis of all GBS isolates.

Genotyping of all the isolates detected seven GBS clusters I, II, III, IV, V, VI, and VII with the following prevalence percentages: 18.4%, 13.2%, 19.4%, 7.9%, 11.8%, 11.8%, and 17.1%, respectively. A dendrogram of the seven clusters of genotypes is shown in Figure 1. The most common cluster was III (19.7%) and the least prevalent was IV (7.9%). Table 1 shows the correlation between GBS clusters and their AR profiles. 

 The data show a prevalence of certain genotype clusters in both tertiary care centers while other genotype clusters were merely confined to a particular tertiary center (Table 2). Similarly, certain AR profiles were prevalent in both centers, whereas others were restricted to one center.

### 3.3. PCR Amplification of the Virulence Genes

PCR detection of the virulence genes showed that *cylE, lmb, scpB*, *bca*, and *rib* genes were positive in 99%, 96.1%, 94.7%, 56.6%, and 33% of the GBS isolates respectively. There was a wide prevalence of the *cylE, lmb*, and *scpB* genes among the total isolates, and hence all 3 genes were evenly distributed among the genotype clusters. Table 3 shows the prevalence of *bca* and *rib* genes among the GBS genotype clusters. Sixty eight isolates out of 76 (89.5%) have either the *bca *or the *rib* gene and 8 isolates out of 76 (10.5%) have neither *bca* nor *rib *detected.

## 4. Discussion

Neonatal GBS infection is a serious complication of the vertical transmission of the bacteria, from the mother to the newborn, at the time of vaginal delivery [1]. This is why universal screening for GBS vaginal colonization in pregnant women near term is recommended [2]. 

 Fortunately, GBS resistant to penicillin has not been reported all around the world, and the drug of choice, in treating the infection, is still penicillin [7, 9]. However, the problem arises in cases, allergic to penicillin, where alternate antimicrobial agents such as CLI and ERY are commonly administered [2]. However, De Azavedo et al. [9] found that 18%, 8%, and >80% of GBS isolates in pregnant women were resistant to ERY, CLI, and TET [9]. In our study, 11.8%, 15.8%, and 86.8% of the GBS isolates, were resistant to ERY, CLI, and TET respectively, indicating percentages similar to previous studies. The prevalence of this relatively high AR percentages, leads to the recommendation of requesting a sensitivity antibiogram for GBS cultured from women allergic to penicillin. 

 Genotyping of all the GBS isolates, utilizing RAPD analysis determined a total of 7 genotype clusters. The most common was genotype cluster III (19.7%) and the least common was IV found in 7.9% of all isolates. 

 A correlation between AR and genotype clusters, showed an association between them (Table 1). Resistance to CHL was restricted to genotype clusters V, VI, and VII, whereas resistance to both CLI and ERY was found only in genotype clusters I, II, and VII. All the GBS isolates resistant to CLI are also resistant to ERY, indicating a correlating pattern of resistance amongst the genes responsible for this phenotype. This can be expected since CLI resistance in GBS is nearly always based on the presence of an *ermB* gene conferring resistance to macrolides, lincosamides, and streptogramin B [27]. 

 An important finding in this study was the correlation between the prevalence of particular genotype clusters with certain AR profiles in a given medical center. This observation denotes that high prevalence of certain genotype clusters resistant to certain antimicrobial agents in a particular medical center entails the practice of caution by extrapolating the AR findings from one medical center to others. 

 Another observation is that* bca* and *rib* genes were not present concomitantly in the same genome. Previous studies noted this observation [28, 29]. Rib protein encoded by the *rib* gene shares several properties with *α*-C protein encoded by *bca* gene. Both proteins are resistant to trypsin digestion and belong to the same family of bacterial surface proteins with repetitive structures showing a 47% identity, their N-terminal sequences are related and are 61% identical to each other [30]. These properties suggest that both proteins may have a common origin. Further investigations are still needed to be performed in order to discover the functional relationship between *bca* and *rib* to determine if they have homologous functions.

## Figures and Tables

**Table 1:** Distribution of antimicrobial resistance profiles among GBS genotype clusters.

AR profile (# of isolates)	A (3)	B (5)	C (1)	D (4)	E (53)	F (10)
Genotype Clusters (# of isolates)						
I (14)	0	2	0	1	8	3
II (10)	0	1	0	0	9	0
III (15)	0	0	1	0	13	1
IV (6)	0	0	0	0	3	3
V (9)	1	0	0	2	6	0
VI (9)	1	0	0	1	5	2
VII (13)	1	2	0	0	9	1

Antimicrobial Resistance (AR) profile: resistance to CLI, ERY, CHL, and TET (A); resistance to CLI, ERY, and TET (B); resistance to CLI and TET (C); resistance to ERY and TET (D); resistance to only TET (E); susceptible to all antibiotics (F).

**Table 2:** Distribution of GBS genotypes in two medical centers.

Medical Center	A (49)	B (27)
Genotype Clusters (# of isolates)		
I (14)	7	7
II (10)	10	0
III (15)	0	15
IV (6)	6	0
V (9)	9	0
VI (9)	4	5
VII (13)	13	0

A: Medical center located in the Western part of Beirut.

B: Medical center located in the Eastern part of Beirut.

**Table 3:** Distribution of *bca* and *rib* virulence genes in GBS genotype clusters.

Genotype Clusters	*bca* gene	*rib* gene
(# of isolates)	# positive (%)	# positive (%)
I (14)	5 (35.7)	6 (42.9)
II (10)	6 (60.0)	4 (40.0)
III (15)	9 (60.0)	5 (33.3)
IV (6)	5 (83.3)	1 (16.7)
V (9)	4 (44.4)	3 (33.3)
VI (9)	7 (77.8)	2 (22.2)
VII (13)	7 (53.8)	4 (30.7)
Total (76)	43 (56.5)	25 (32.9)

## References

[1] Persson E, Berg S, Trollfors B (2004). Serotypes and clinical manifestations of invasive group B steptococcal infections in western Sweden 1998–2001. *Clinical Microbiology and Infection*.

[2] Schrag S, Gorwitz R, Fultz-Butts K (2002). Prevention of perinatal group B streptococcal disease. Revised guidelines from CDC. *Morbidity and Mortality Weekly Report. Recommendations and Reports*.

[3] Baltimore RS (2007). Consequences of prophylaxis for group B streptococcal infections of the neonate. *Seminars in Perinatology*.

[4] Hansen SM, Uldbjerg N, Kilian M (2004). Dynamics of *Streptococcus agalactiae* colonization in women during and after pregnancy and in their infants. *Journal of Clinical Microbiology*.

[5] Moore MR, Schrag SJ, Schuchat A (2003). Effects of intrapartum antimicrobial prophylaxis for prevention of group-B-streptococcal disease on the incidence and ecology of early-onset neonatal sepsis. *Lancet Infectious Diseases*.

[6] Zhang GW, Kotiw M, Daggard G (2002). A RAPD-PCR genotyping assay which correlates with serotypes of group B streptococci. *Letters in Applied Microbiology*.

[7] Persson E, Berg S, Bergseng H (2008). Antimicrobial susceptibility of invasive group B streptococcal isolates from south-west Sweden 1988–2001. *Scandinavian Journal of Infectious Diseases*.

[8] Nagano N, Nagano Y, Kimura K, Tamai K, Yanagisawa H, Arakawa Y (2008). Genetic heterogeneity in pbp genes among clinically isolated group B streptococci with reduced penicillin susceptibility. *Antimicrobial Agents and Chemotherapy*.

[9] De Azavedo JCS, McGavin M, Duncan C (2001). Prevalence and mechanisms of macrolide resistance in invasive and noninvasive group B streptococcus isolates from Ontario, Canada. *Antimicrobial Agents and Chemotherapy*.

[10] Marques MB, Kasper DL, Pangburn MK, Wessels MR (1992). Prevention of C3 deposition by capsular polysaccharide is a virulence mechanism of type III group B streptococci. *Infection and Immunity*.

[11] Beckmann C, Waggoner JD, Harris TO (2002). Identification of novel adhesins from group B streptococci by use of phage display reveals that C5a peptidase mediates fibronectin binding. *Infection and Immunity*.

[12] Bolduc GR, Baron MJ, Gravekamp C (2002). The alpha C protein mediates internalization of group B *Streptococcus* within human cervical epithelial cells. *Cellular Microbiology*.

[13] Spellerberg B, Rozdzinski E, Martin S (1999). Lmb, a protein with similarities to the LraI adhesin family, mediates attachment of *Streptococcus agalactiae* to human laminin. *Infection and Immunity*.

[14] Doran KS, Liu GY, Nizet V (2003). Group B streptococcal *β*-hemolysin/cytolysin activates neutrophil signaling pathways in brain endothelium and contributes to development of meningitis. *Journal of Clinical Investigation*.

[15] Stålhammar-Carlemalm M, Stenberg L, Lindahl G (1993). Protein rib: a novel group B streptococcal cell surface protein that confers protective immunity and is expressed by most strains causing invasive infections. *Journal of Experimental Medicine*.

[16] Lindahl G, Stålhammar-Carlemalm M, Areschoug T (2005). Surface proteins of *Streptococcus agalactiae* and related proteins in other bacterial pathogens. *Clinical Microbiology Reviews*.

[17] Areschoug T, Stålhammar-Carlemalm M, Karlsson I, Lindahl G (2002). Streptococcal *β* protein has separate binding sites for human factor H and IgA-Fc. *The Journal of Biological Chemistry*.

[18] Schubert A, Zakikhany K, Schreiner M, Frank R, Spellerberg B, Eikmanns BJ, Reinscheid DJ (2002). A fibrinogen receptor from group B *Streptococcus* interacts with fibrinogen by repetitive units with novel ligand binding sites. *Molecular Microbiology*.

[19] Tenenbaum T, Bloier C, Adam R, Reinscheid DJ, Schroten H (2005). Adherence to and invasion of human brain microvascular endothelial cells are promoted by fibrinogen-binding protein FbsA of *Streptococcus agalactiae*. *Infection and Immunity*.

[20] Gutekunst H, Eikmanns BJ, Reinscheid DJ (2004). The novel fibrinogen-binding protein FbsB promotes *Streptococcus agalactiae* invasion into epithelial cells. *Infection and Immunity*.

[21] Harris TO, Shelver DW, Bohnsack JF, Rubens CE (2003). A novel streptococcal surface protease promotes virulence, resistance to opsonophagocytosis, and cleavage of human fibrinogen. *Journal of Clinical Investigation*.

[22] Clinical and Laboratory Standards Institute (2005). Performance standards for antimicrobial susceptibility testing; fifteenth informational supplement. *CLSI document*.

[23] Bergseng H, Bevanger L, Rygg M (2007). Real-time PCR targeting the sip gene for detection of group B *Streptococcus* colonization in pregnant women at delivery. *Journal of Medical Microbiology*.

[24] Smith TC, Roehl SA, Pillai P (2007). Distribution of novel and previously investigated virulence genes in colonizing and invasive isolates of *Streptococcus agalactiae*. *Epidemiology and Infection*.

[25] Duarte RS, Bellei BC, Miranda OP (2005). Distribution of antimicrobial resistance and virulence-related genes among Brazilian group B streptococci recovered from bovine and human sources. *Antimicrobial Agents and Chemotherapy*.

[26] Dmitriev A, Suvorov A, Shen AD, Yang YH (2004). Clinical diagnosis of group B streptococci by scpB gene based PCR. *Indian Journal of Medical Research*.

[27] Jenssen WD, Thakker-Varia S, Dubin DT, Weinstein MP (1987). Prevalence of macrolides-lincosamides-streptogramin B resistance and erm gene classes among clinical strains of staphylococci and streptococci. *Antimicrobial Agents and Chemotherapy*.

[28] Kong F, Gowan S, Martin D, James G, Gilbert GL (2002). Molecular profiles of group B streptococcal surface protein antigen genes: relationship to molecular serotypes. *Journal of Clinical Microbiology*.

[29] Lachenauer CS, Creti R, Michel JL, Madoff LC (2000). Mosaicism in the *α*-like protein genes of group B streptococci. *Proceedings of the National Academy of Sciences of the United States of America*.

[30] Wästfelt M, Stålhammar-Carlemalm M, Delisse A-M, Cabezon T, Lindahl G (1996). Identification of a family of streptococcal surface proteins with extremely repetitive structure. *The Journal of Biological Chemistry*.

